# A57 VANISHING PANCREAS: HIPPO-MEDIATED FOCAL REPLACEMENT OF THE EXOCRINE PANCREAS WITH ADIPOSE TISSUE

**DOI:** 10.1093/jcag/gwac036.057

**Published:** 2023-03-07

**Authors:** J Messina-Pacheco, A Gregorieff

**Affiliations:** 1 Department of Pathology, McGill University; 2 Cancer Research Program, Research Institute - McGill University Health Centre; 3 McGill Regenerative Medicine Network, Montreal, Canada

## Abstract

**Background:**

The pancreas exhibits remarkable inherent cellular plasticity in response to injury. To prevent inflammatory injury or death, acinar cells can undergo transient acinar-to-ductal metaplasia (ADM) by suspending normal cell functions and adopting characteristics of ductal cells. However, persistent ADM in the setting of chronic pancreatitis predisposes to pancreatic cancer. Less frequently, acinar cells have also been found to undergo acinar-to-adipocyte transdifferentiation, but the mechanisms and clinical significance of this process are largely unknown. Recent studies have identified that the Hippo signaling pathway and its effectors are vital for pancreatic development and function.

**Purpose:**

YAP is highly expressed in normal pancreatic ducts and transiently in acinar cells undergoing ADM, suggesting a dual role for YAP in (1) the homeostatic maintenance of pancreatic ductal cells, and (2) the regenerative response to injury in acinar cells. However, little is known about the cell type-specific effects of YAP/TAZ on pancreas homeostasis and regeneration.

**Method:**

We investigated the homeostatic functions of Yap and Taz in the pancreas by conditionally ablating *Yap/Taz* in both acinar cells and ductal cells using the previously described Clu^CreERT^ mouse line. We also established a pancreatic ductal cell-derived organoid system. The efficiency of *in vitro* Cre recombinase induction was confirmed in Yap^fl/fl;^Taz^fl/fl^;Clu^Cre-ERT^;LSL-tdTomato (YTKO) ductal organoids.

**Result(s):**

We observed severe atrophy and a pancreatitis-like phenotype in the pancreata of YTKO mice following tamoxifen induction. At later time-points, YTKO pancreata were progressively remodeled – the exocrine pancreas was almost entirely replaced by adipose tissue and large hyperplastic ductal structures. We will perform further lineage tracing experiments to determine whether infiltrating adipose cells derive directly from transdifferentiating acinar cells. YTKO pancreatic ductal organoids exhibited disrupted survival and proliferation, evidenced by increased expression of cleaved Caspase3 and decreased EdU incorporation compared to vehicle-treated controls.

**Conclusion(s):**

Although some flexibility in cell fate potential is beneficial for the regenerative capacity of the pancreas, dramatic changes in cellular identity can have disastrous consequences. Overall, this study revealed that disruptions in Hippo signaling in the adult murine pancreas led to failure of regeneration and the complete remodeling of the exocrine pancreas, and has shed light on the previously uncharacterized role of Hippo signaling in acinar-to-adipocyte transdifferentiation. The potential contribution of fatty infiltration of the pancreas to the pathogenesis of diabetes mellitus and pancreatic cancer merits further exploration.

**Please acknowledge all funding agencies by checking the applicable boxes below:**

CIHR, Other

**Please indicate your source of funding;:**

Fonds de recherche du Quebec - Sante (FRQS)

**Disclosure of Interest:**

None Declared

CELLULAR & MOLECULAR GASTROENTEROLOGY

